# Effects of comprehensive nursing interventions on pain, recovery, and sexual quality of life in patients with adenomyosis: a prospective cohort study

**DOI:** 10.3389/fpubh.2026.1870586

**Published:** 2026-09-03

**Authors:** Wenjuan Zhu, Shuzhen Gan, Liu Yang

**Affiliations:** 1 West Anhui Health Vocational College, Lu'an, Anhui, China; 2 Department of Obstetrics, Ganzhou People's Hospital, Ganzhou, China; 3 Department of Reproductive Medicine, Ganzhou People's Hospital, Ganzhou, China

**Keywords:** adenomyosis, comprehensive nursing, mental health, pain, sexual quality of life

## Abstract

**Objective:**

To explore the effectiveness of comprehensive nursing intervention in patients with adenomyosis and its impact on pain and sexual quality of life.

**Methods:**

This prospective, non-randomized study included 120 patients with adenomyosis who received treatment at Ganzhou People's Hospital between November 2022 and March 2024. All patients underwent total hysterectomy. Patients with concurrent endometriosis were not excluded; however, baseline characteristics between groups were comparable (*P* > 0.05 for all variables). The patients were divided into a control group (*n* = 60) and a study group (*n* = 60) based on the timing of their hospitalization relative to a new institutional nursing protocol. The control group received routine nursing care, while the study group underwent comprehensive nursing intervention in addition to routine care. Pain scores (VAS), sexual quality of life (FSFI scale), psychological status (SAS, SDS), quality of life (SF-36), and nursing satisfaction were evaluated prospectively by trained research staff at baseline (admission) and at discharge.

**Results:**

There were no significant differences in baseline data between the two groups (*P* > 0.05). Post-intervention, the study group had lower VAS scores compared to the control group (*P* < 0.05). The study group also showed reduced postoperative bleeding, earlier ambulation, shorter time to first flatus, and shorter hospital stays compared to the control group (*P* < 0.05). Additionally, FSFI scores, including all subcategories and the total score, were higher in the study group (*P* < 0.05). The study group exhibited lower post-intervention SAS and SDS scores (*P* < 0.05) and higher SF-36 scores (*P* < 0.05) than the control group. Nursing satisfaction was significantly higher in the study group compared to the control group (*P* < 0.05).

**Conclusion:**

Comprehensive nursing intervention provides multidimensional support tailored to the needs of patients, alleviating tension and fear, and promoting an optimal treatment state. It effectively reduces pain and improves sexual quality of life in patients with adenomyosis, making it worthy of clinical application. Given the prospective, non-randomized, single-center design of this study, the findings should be interpreted as demonstrating associations rather than causal relationships.

## 1 Introduction

Adenomyosis is a common benign gynecological condition characterized by the infiltration of endometrial glands and stroma into the myometrium, leading to myometrial hyperplasia and pathological remodeling. The disease predominantly affects women of reproductive age, with primary symptoms including progressive dysmenorrhea, irregular vaginal bleeding, menorrhagia, and chronic pelvic pain—all of which significantly impair patients' physical functioning, psychological wellbeing, and overall quality of life (1, 2). Pain is among the most prominent and distressing symptoms, often correlating with the depth and extent of myometrial involvement. In severe cases, it can disrupt daily activities, reduce work productivity, and contribute to anxiety or depression. Moreover, adenomyosis frequently compromises sexual health, manifesting as decreased libido, dyspareunia, and reduced satisfaction with intimate relationships. This dual physiological and psychological burden not only undermines treatment adherence but may also exacerbate long-term declines in health-related quality of life. In some instances, prolonged disease progression or suboptimal management can further impair fertility, intensifying emotional distress and social strain (3, 4).

Notably, adenomyosis often coexists with endometriosis. Epidemiological studies report that 15% to 40% of patients with adenomyosis also have endometriosis, and recent retrospective cohort analyses based on histopathological confirmation have identified concurrent endometriosis in as many as 71.0% of cases. Patients with both conditions tend to present with more severe symptoms and distinct demographic or clinical profiles compared to those with isolated adenomyosis (5). This high rate of comorbidity underscores the complexity of symptom presentation and highlights the need to consider both entities when evaluating disease burden and therapeutic responses.

Despite significant advances in imaging technologies and heightened clinical awareness—leading to improved diagnostic accuracy—the effective management of adenomyosis remains a persistent challenge, particularly in complex or comorbid cases. In this context, comprehensive nursing intervention, a multidimensional and patient-centered care model that integrates physical, psychological, and social support, has gained increasing attention. Emerging evidence suggests that such interventions can positively influence pain control, mental health, and overall quality of life in chronic gynecological conditions (6, 7). However, systematic research specifically focused on patients with adenomyosis is still limited.

Against this backdrop, this prospective study aims to evaluate the impact of comprehensive nursing intervention on pain alleviation, improvement in sexual quality of life, and enhancement of nursing satisfaction among women diagnosed with adenomyosis at a single tertiary hospital. The findings are intended to provide both theoretical insights and practical guidance for optimizing holistic nursing care in this underserved patient population.

## 2 Materials and methods

### 2.1 General information

This was a prospective cohort study with non-randomized allocation based on chronological admission cohorts, conducted at Ganzhou People‘s Hospital between November 2022 and March 2024. A total of 120 women diagnosed with adenomyosis who were scheduled to undergo total hysterectomy were consecutively enrolled. Inclusion criteria required a preoperative diagnosis confirmed by transvaginal ultrasound or pelvic MRI, with final pathological verification post-hysterectomy. Patients were excluded if they had incomplete data, severe comorbidities (e.g., cardiac, hepatic, or renal failure), psychiatric disorders affecting communication, or refused surgery. Patients with concurrent endometriosis were not excluded given its high co-occurrence with adenomyosis in clinical practice (8–11).

Patients were allocated to groups based on the timing of hospital admission relative to the implementation of a new institutional nursing protocol. Patients admitted before January 2023 received routine postoperative care (control group, *n* = 60), while those admitted from January 2023 onward received the comprehensive nursing intervention in addition to routine care (study group, *n* = 60). Baseline characteristics were compared between groups to assess comparability. The non-randomized, chronological allocation may introduce selection bias, which is addressed in the Discussion section.

The study protocol was approved by the Ethics Committee of Ganzhou People‘s Hospital (Approval No.: GZ490-2021) on June 1, 2021, with a protocol amendment approved in December 2022. The study adhered to the principles of the Declaration of Helsinki and was conducted within the approval validity period (June 1, 2021 to March 31, 2024).

Given the mixed design of this study, the consent procedures differed between groups:

(1) For the control group (admitted before January 2023, *n* = 60): These patients had already been discharged before the study amendment and informed consent procedures were implemented. However, the outcome measures (VAS, FSFI, SAS, SDS, and SF-36 scores) had been routinely documented as part of the department‘s standard clinical nursing assessment protocol for hysterectomy patients during their hospitalization. Following the protocol amendment, trained research nurses retrospectively extracted these existing data from anonymized medical records at standardized time points (admission and discharge). The requirement for written informed consent was formally waived by the Ethics Committee for this historical cohort, as obtaining consent was not practicable and all data were anonymized prior to analysis.
(2) For the study group (admitted from January 2023 onward, *n* = 60): These patients were enrolled prospectively after the protocol amendment was approved. Prior to surgery, trained research nurses provided detailed verbal and written explanations of the study protocol to all potential participants, covering the purpose, procedures, potential risks, benefits, and the voluntary nature of participation. All participants were explicitly informed that they could withdraw from the study at any time without any negative impact on their standard medical care. Written informed consent was obtained from each patient in this prospective cohort before surgery, following these explanations. The same standardized instruments (VAS, FSFI, SAS, SDS, SF-36) were administered at admission and discharge by the same trained research nurses to ensure comparability across groups.

### 2.2 Nursing care documentation

Nursing interventions were prospectively delivered and documented by the clinical nursing staff according to the assigned group.

#### 2.2.1 Control group

Patients received conventional postoperative nursing care, which included routine vital sign monitoring, wound observation, and basic hygiene assistance.

#### 2.2.2 Study group

Patients received the comprehensive nursing care bundle, a structured protocol officially adopted by the hospital in January 2023 for adenomyosis patients undergoing hysterectomy. The bundle consisted of the following six components:

(1) Preoperative education addressing common misconceptions about hysterectomy, with specific emphasis on its limited impact on ovarian endocrine function and sexual health;
(2) Individualized psychological support involving family participation and strategies to alleviate anxiety;
(3) Enhanced postoperative pain management, including scheduled VAS assessments and timely adjustment of analgesic regimens;
(4) Proactive complication prevention measures, such as regular perineal cleaning to reduce urinary tract infection risk, encouragement of early ambulation to prevent venous stasis, and individualized fluid intake guidance;
(5) Instruction in pelvic floor muscle exercises and bladder retraining techniques; and
(6) Detailed discharge counseling, particularly advising abstinence from sexual intercourse for at least 2 months postoperatively.

### 2.3 Outcome measures

Outcome measures were assessed prospectively by trained research nurses at two time points: baseline (upon admission) and at discharge.

Pain intensity was assessed using the Visual Analog Scale (VAS; 0 = no pain, 10 = worst imaginable pain).

Recovery indicators included estimated postoperative blood loss, time to first ambulation, time to first flatus, and total hospital stay (days).

Sexual quality of life at discharge was evaluated using the validated Chinese version of the Female Sexual Function Index (FSFI), covering desire, arousal, lubrication, orgasm, satisfaction, and dyspareunia (total score range: 2–36; higher scores indicate better function).

Psychological status was assessed via the Self-Rating Anxiety Scale (SAS) and Self-Rating Depression Scale (SDS); lower scores reflect better emotional wellbeing.

Health-related quality of life was measured using the SF-36 questionnaire, summarized into physical and mental component scores.

#### 2.3.1 Nursing satisfaction

A self-designed nursing satisfaction questionnaire was administered to assess patient satisfaction at discharge. The questionnaire was developed based on a review of existing patient satisfaction instruments and clinical nursing quality indicators. Content validity was assessed by a panel of three senior nursing experts, and face validity was confirmed through a pilot test with 10 patients (not included in the final study sample). The questionnaire demonstrated acceptable internal consistency (Cronbach's α = 0.82). It included items on communication, responsiveness, and overall care quality, with responses categorized into three levels: “satisfied,” “somewhat satisfied,” and “dissatisfied.” For statistical analysis, responses of “satisfied” and “somewhat satisfied” were combined into a single “satisfied” category, and the overall satisfaction rate was calculated as the proportion of patients in this combined category out of the total number of patients in each group.

### 2.4 Statistical analysis

Data were analyzed using SPSS version 26.0 (IBM Corp., Armonk, NY, USA). Continuous variables were presented as mean ± standard deviation (x ± SD) and compared between groups using independent-samples *t*-tests. Categorical variables were expressed as frequencies (%) and analyzed with chi-square (χ^2^) tests. A two-sided *P*-value < 0.05 was considered statistically significant. Graphical presentations were generated using GraphPad Prism 8.0.

## 3 Results

### 3.1 Baseline data

The control group consisted of 60 patients aged 28–55 years, with a mean age of (42.88 ± 3.68) years and a disease duration of 4–23 months, averaging (11.28 ± 2.71) months. Their educational levels were as follows: 12 patients with junior high school education or below, 29 with high school education, and 19 with a college degree or above. The study group included 60 patients aged 28–55 years, with a mean age of (42.97 ± 3.45) years and a disease duration of 4–23 months, averaging (11.17 ± 2.38) months. Their educational levels were as follows: 11 patients with junior high school education or below, 28 with high school education, and 21 with a college degree or above. The duration of medical treatment for pain prior to admission was also comparable between the two groups (control: 6.52 ± 3.14 months; study: 6.38 ± 2.97 months; *P* = 0.801). There was no significant difference in baseline data between the two groups, indicating comparability (*P* > 0.05). Refer to Table 1.

**Table 1:** Comparison of baseline data between the two groups.

Item	Control group	Research group	t/χ^2^	*P*
Number of cases	60	60	-	-
Age (years)—Range	28–55	28–55	-	-
Age (years)—Mean	42.88 ± 3.68	42.97 ± 3.45	0.138	0.89
Disease duration (months)—Range	46,135	46,135	-	-
Disease duration (months)—Mean	11.28 ± 2.71	11.17 ± 2.38	0.236	0.814
Duration of pain treatment (months)—Mean	6.52 ± 3.14	6.38 ± 2.97	0.252	0.801
Degree of education—Junior high school and below	12	11	-	-
Degree of education - High school	29	28	-	-
Degree of education—Junior college or above	19	21	-	-
Surgical approach (abdominal/laparoscopic)	38/22	35/25	0.349	0.555

### 3.2 Pain levels

**Figure 1:**
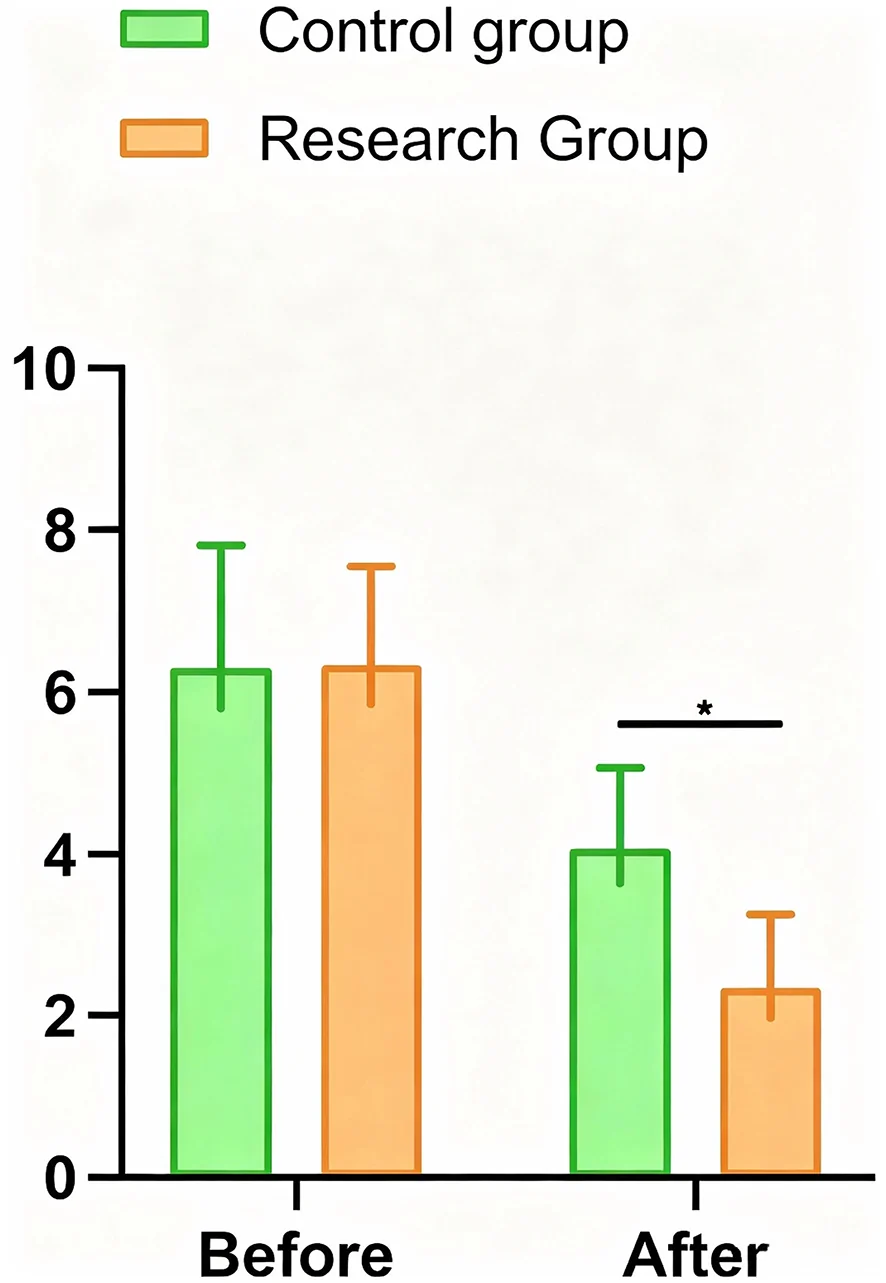
Comparison of VAS scores before and after care between the two groups. * indicates a significant difference between the two groups, *P* < 0.05.

The post-care VAS score of the study group (2.34 ± 0.93) was significantly lower than that of the control group (4.05 ± 1.02), with *P* < 0.05. Refer to Figure 1.

### 3.3 Recovery indicators

The study group demonstrated significantly lower postoperative blood loss, earlier ambulation time, earlier first flatus, and shorter hospital stay compared to the control group (*P* < 0.05). Refer to Table 2.

**Table 2:** Comparison of recovery indicators between the two groups.

Item	Control group	Research group	*t*	*P*
Number of cases	60	60	_−_	_−_
Postoperative bleeding volume (ml)	151.65 ± 33.15	100.89 ± 28.38	9.010	< 0.001
Bedtime (h)	38.14 ± 8.14	18.08 ± 5.22	16.069	< 0.001
First exhaust time (h)	28.11 ± 5.94	15.55 ± 4.98	12.551	< 0.001
Hospitalization time (d)	6.88 ± 0.94	4.22 ± 0.49	19.437	< 0.001

### 3.4 Sexual quality of life

**Figure 2:**
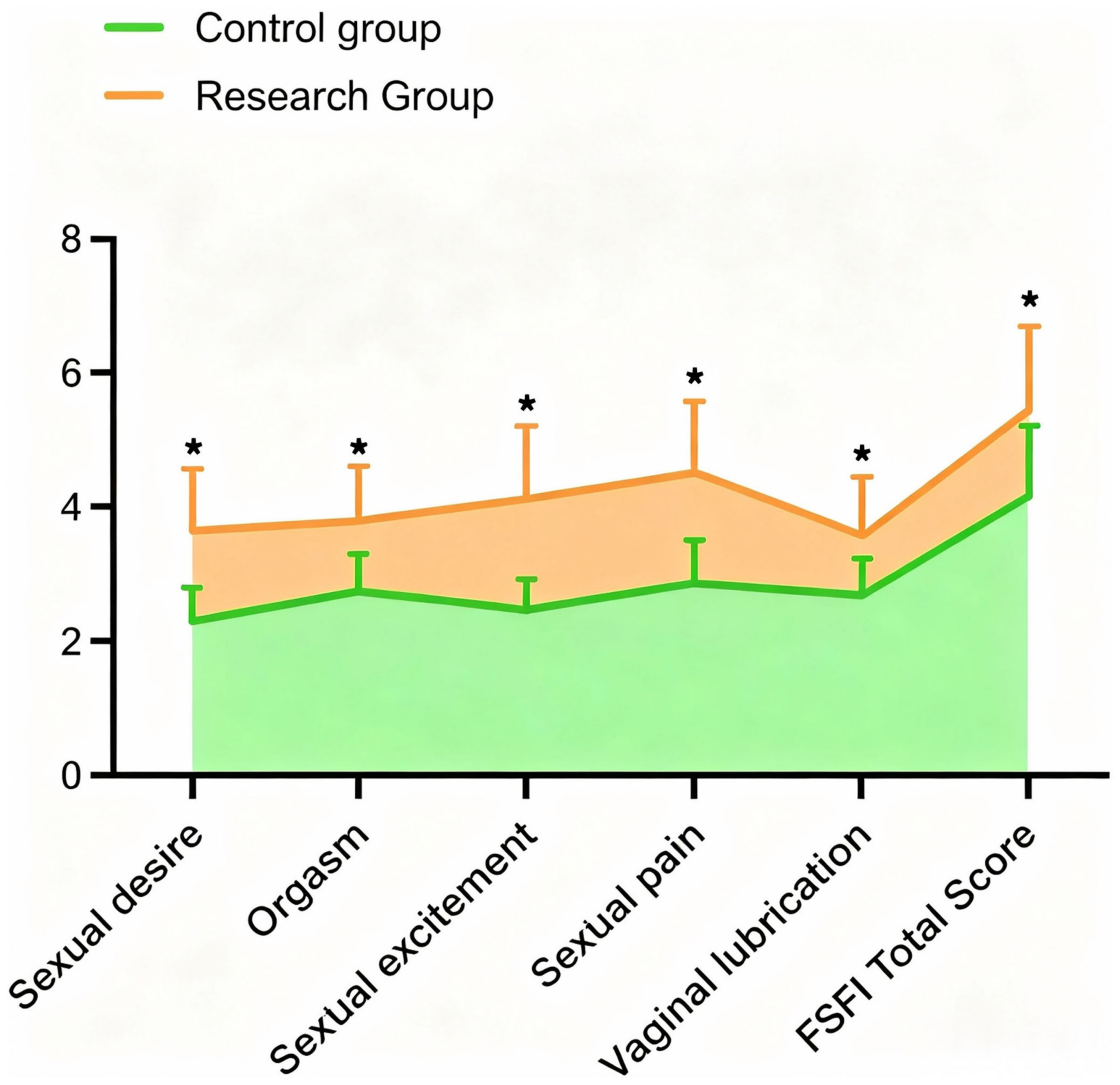
Comparison of FSFI subcategory and total scores between the two groups. * indicates a significant difference between the two groups, *P* < 0.05.

The FSFI scores, including all subcategories and the total score, were significantly higher in the study group (3.61 ± 0.94, 3.77 ± 0.85, 4.08 ± 1.11, 4.47 ± 1.08, 3.56 ± 0.89, 5.41 ± 1.23) compared to the control group (2.28 ± 0.51, 2.71 ± 0.56, 2.45 ± 0.47, 2.83 ± 0.65, 2.66 ± 0.56, 4.12 ± 1.07), with *P* < 0.05. Refer to Figure 2.

### 3.5 Negative emotions

**Figure 3:**
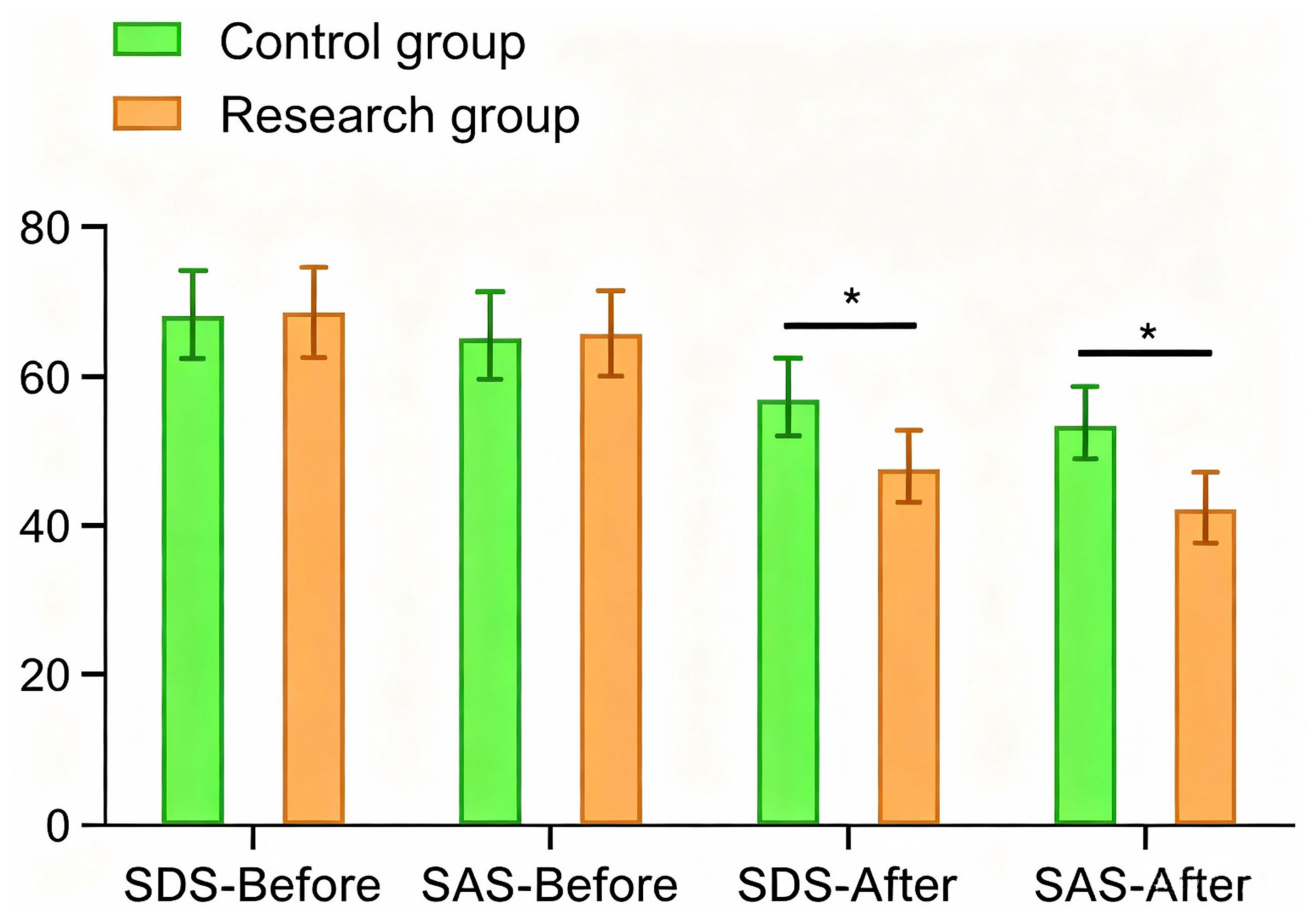
Comparison of SAS and SDS scores between the two groups. * indicates a significant difference between the two groups, *P* < 0.05.

The SAS and SDS scores of the study group (47.56 ± 5.29, 42.18 ± 5.11) were significantly lower than those of the control group (56.56 ± 5.89, 53.14 ± 5.47), with *P* < 0.05. Refer to Figure 3.

### 3.6 Quality of life

**Figure 4:**
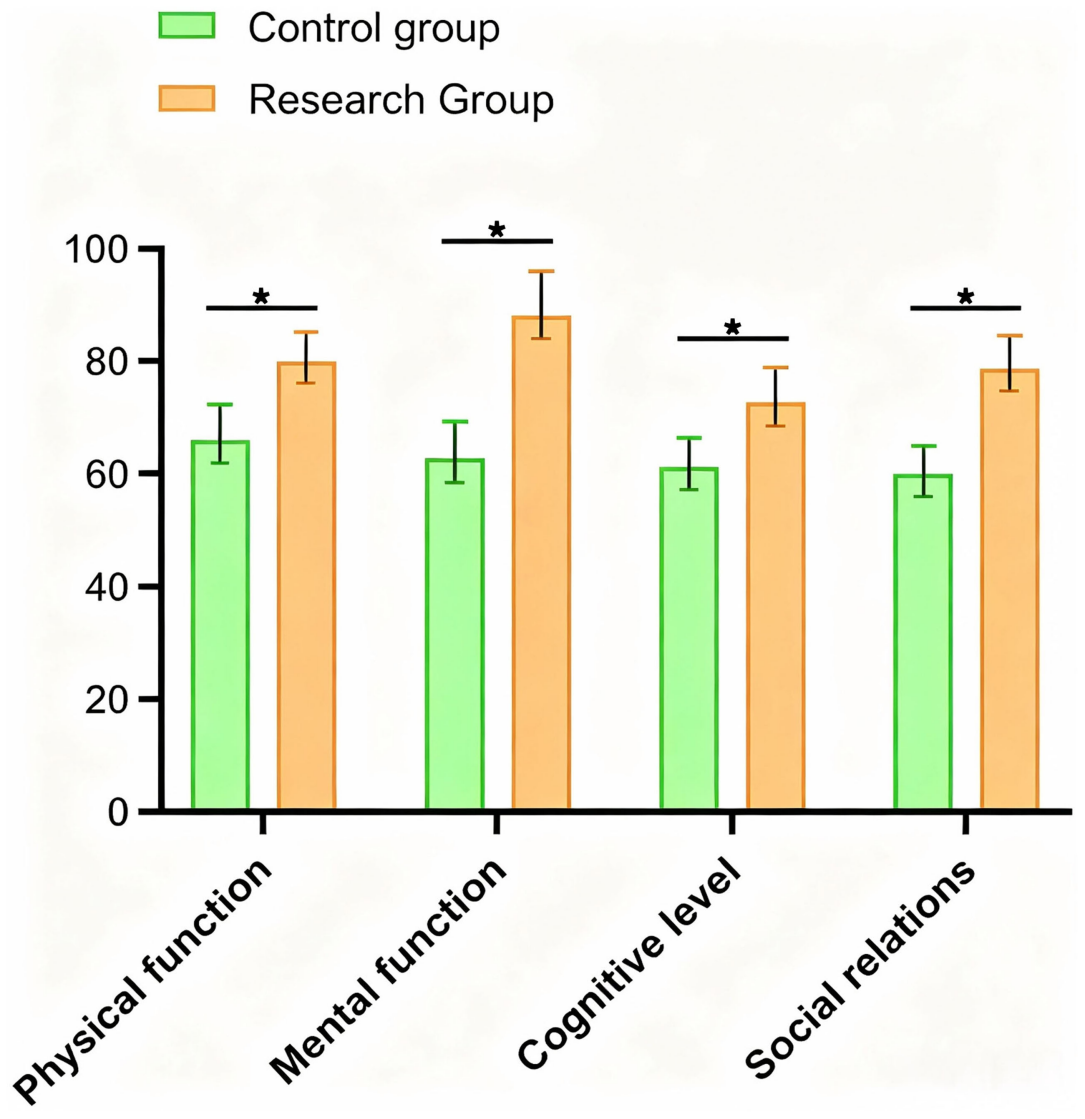
Comparison of SF-36 postoperative scores between the two groups. * indicates a significant difference between the two groups, *P* < 0.05.

The SF-36 scores across all domains for the study group (80.14 ± 5.69, 87.96 ± 8.37, 72.69 ± 6.33, 79.14 ± 5.63) were significantly higher than those of the control group (65.26 ± 6.61, 62.34 ± 6.99, 61.08 ± 5.17, 60.11 ± 4.56), with *P* < 0.05. Refer to Figure 4.

### 3.7 Nursing satisfaction

The overall nursing satisfaction rate (combining satisfied and somewhat satisfied) in the study group was 98.33% (59/60), which was significantly higher than the 83.33% (50/60) observed in the control group (χ^2^ = 8.107, *P* = 0.004). Refer to Table 3.

**Table 3:** Comparison of nursing satisfaction between the two groups.

Item	Satisfied	Somewhat satisfied	Dissatisfied	Overall satisfaction rate
Control Group (*n* = 60)	38 (63.33)	12 (20.00)	10 (16.67)	50 (83.33)
Research Group (*n* = 60)	57 (95.00)	2 (3.33)	1 (1.67)	59 (98.33)
χ^2^	-	-	-	8.107
*P*	-	-	-	0.004

## 4 Discussion

Adenomyosis, also known as intrinsic endometriosis, is a benign condition characterized by the invasion of endometrial tissue into the uterine myometrium, leading to pathological changes. It predominantly occurs in multiparous women aged 30–50 years, with an incidence rate ranging from 10% to 47%, and is particularly common in women nearing menopause. Although the exact pathogenesis of adenomyosis remains unclear, studies suggest that cesarean section scars and uterine curettage may act as triggering factors. During surgical treatment, an enlarged uterus often reveals fresh or old hemorrhages within the myometrium, indicating that ectopic endometrial tissue contributes to the pathology (12–14). Surgery is a commonly used treatment for adenomyosis and has proven to be effective. However, it may also impact patients' physical and psychological wellbeing, as perioperative periods are often accompanied by stress reactions such as tension and anxiety, which can affect surgical outcomes and postoperative recovery (15–17). Traditional nursing care typically focuses on basic care and medication guidance. While these measures may alleviate symptoms to some extent, they lack targeted and comprehensive interventions, making it difficult to address the multifaceted needs of patients (18–20). Our findings align with a growing body of international evidence highlighting the critical role of holistic perioperative support in gynecological surgery. The results of this study demonstrate that comprehensive nursing interventions not only effectively reduce pain and improve quality of life but also shorten postoperative recovery time and enhance nursing satisfaction, showcasing their significant advantages.

The findings revealed that patients in the study group had significantly lower VAS scores post-care compared to the control group (*P* < 0.05), indicating the superiority of comprehensive nursing in pain management. By incorporating psychological counseling, non-pharmacological interventions (e.g., hot compresses, massage), and standardized pharmacological pain management, comprehensive care helped alleviate postoperative pain. This multi-layered intervention approach significantly improved patients' pain experiences, enhancing overall care quality. Moreover, the study group exhibited significantly lower postoperative bleeding volume, earlier mobilization and first flatus times, and shorter hospital stays compared to the control group (*P* < 0.05), reflecting the positive impact of comprehensive nursing on recovery. These outcomes may be attributed to personalized postoperative guidance, enhanced monitoring, and early mobilization interventions. By promoting blood circulation, reducing postoperative complications, and accelerating recovery, comprehensive nursing effectively shortened hospitalization durations, alleviating patients' financial and psychological burdens. These recovery benefits are comparable to those reported in recent Enhanced Recovery After Surgery (ERAS) protocols in gynecological surgery, where structured perioperative pathways have consistently demonstrated reduced time to first flatus and shortened hospitalization (21, 22). In treating adenomyosis, surgical approaches such as total hysterectomy are common options. However, as an invasive treatment, surgery can trigger psychological and physiological stress responses, potentially negatively affecting therapeutic outcomes (23, 24). Therefore, implementing effective nursing interventions is crucial to ensuring patients' physical and mental wellbeing. Comprehensive nursing, as a systematic and efficient care model, revolutionizes the traditional disease-centered approach by focusing on designing personalized care plans tailored to patients' individual circumstances, thereby enhancing care outcomes. It embodies the “patient-centered” philosophy by emphasizing preoperative health education to enhance patients' understanding of their condition and the surgery, alleviate negative emotions, and improve treatment compliance, ensuring the procedure's success. Additionally, comprehensive nursing prioritizes personal hygiene and infection prevention, further improving care quality, alleviating pain, and promoting postoperative recovery.

In China, traditional cultural and societal influences often lead many spouses to feel embarrassed when discussing sensitive topics. This has resulted in a lack of sufficient attention to the care of patients with adenomyosis, especially after total hysterectomy. To effectively address this issue, implementing targeted psychological care is particularly important. Incorporating sexual knowledge and techniques into health education as a fundamental part of the nursing intervention can significantly alleviate patients‘ postoperative fears. During the first 3 months post-surgery, some patients and their families may still worry about incision dehiscence, infection, pain during intercourse, or vaginal dryness, which can affect sexual quality. However, after a period of adjustment, typically around 6 months postoperatively, patients' sexual quality of life generally improves compared to pre-surgery levels. This observation is consistent with recent high-quality evidence. A 2023 systematic review and meta-analysis by Dedden et al. (25) in The Journal of Sexual Medicine, which analyzed data from over 1,000 women, concluded that hysterectomy is not inherently detrimental to sexual function. Instead, any initial decline is often transient and primarily driven by psychological concerns rather than anatomical changes. More recently, a 2025 study focusing specifically on sexual quality of life after hysterectomy in benign gynecological conditions reported that in patients with adenomyosis, postoperative arousal and orgasm scores improved significantly compared with preoperative levels, further supporting the notion that hysterectomy itself need not impair sexual function when appropriate perioperative support is provided (26). Studies indicate that the impact of hysterectomy on women's sexual quality of life mainly stems from psychological concerns, referred to as psychogenic sexual dysfunction, clinically known as “post-hysterectomy syndrome.” This psychological barrier directly contributes to a decline in patients' sexual quality of life. However, with proactive health education, psychological care, and emotional support, these issues can be effectively managed and alleviated. Therefore, providing synchronous education to both patients and their spouses during the total hysterectomy process is crucial for postoperative recovery and improving quality of life (27–29). The findings of this study also highlight this point. Patients in the study group scored significantly higher on all domains and the total score of the FSFI (Female Sexual Function Index) than the control group (*P* < 0.05), indicating that comprehensive nursing intervention had a remarkable effect on improving patients' sexual quality of life. Providing scientific guidance and psychological support for sexual function issues during the nursing process eased patients' concerns about sexual activities and helped them gradually regain normal sexual function.

The SAS (Self-Rating Anxiety Scale) and SDS (Self-Rating Depression Scale) scores of patients in the study group were significantly lower than those in the control group (*P* < 0.05), demonstrating the vital role of comprehensive nursing in improving patients' psychological wellbeing. Psychological counseling and health education helped patients better understand their condition and surgery, reducing preoperative anxiety and postoperative depression, and promoting mental health recovery, consistent with previous research findings. While surgery and medication remain traditional treatment methods, hysterectomy is the primary treatment for patients with severe symptoms, no fertility requirements, or resistance to drug therapy. However, hysterectomy can trigger stress responses in patients, significantly affecting their endocrine system, leading to negative emotions, and reducing treatment effectiveness. The conventional nursing approach for total hysterectomy in adenomyosis is often inadequate, yielding suboptimal outcomes and failing to meet patients' expectations for care quality. Therefore, strengthening nursing interventions, improving patient compliance, and providing high-quality care are of great necessity (30, 31).

Comprehensive nursing intervention is a novel care model grounded in the holistic “biological-psychological-social” framework. It aims to formulate personalized nursing measures based on scientific theories, clinical diagnosis, and the patient's actual circumstances. This model integrates psychological care, disease knowledge education, and nutritional support to help patients prepare mentally before surgery and secure comprehensive support from family and society. By doing so, it alleviates the psychological and physiological stress responses caused by surgery and encourages patients to actively participate in treatment and care (32, 33).

Relaxation interventions help relieve patients' tension and anxiety, enabling them to maintain an optimal state preoperatively. Postoperatively, comprehensive nursing intervention reduces discomfort and complications through effective care measures, thereby enhancing surgical outcomes and improving patients' quality of life. This was evident in the current study, where the SF-36 scores of the study group were significantly higher than those of the control group after nursing care (*P* < 0.05). This finding underscores the significant role of comprehensive nursing in improving patients' quality of life, likely attributed to its holistic support in psychological care, physical rehabilitation, and guidance on daily activities. Additionally, the study group showed significantly higher nursing satisfaction than the control group (*P* < 0.05), reflecting patients' recognition of comprehensive nursing interventions. High satisfaction not only indicates the quality of nursing services but also strengthens patients' treatment adherence.

It is important to acknowledge that, while our results are promising, this study has inherent limitations due to its prospective, non-randomized, single-center design. The observed benefits should therefore be interpreted as an association between comprehensive nursing care and improved outcomes, rather than definitive proof of causation. Several potential sources of bias and confounding warrant consideration. First, the chronological (non-random) allocation of patients means the control and intervention groups were treated in different time periods (pre- vs. post-January 2023). Concurrent changes in hospital policies, staff training, surgical techniques, or other perioperative protocols—unrelated to the nursing intervention—may have contributed to the observed differences. Second, although baseline pain treatment duration was comparable and measurable covariates were adjusted for, unmeasured confounders such as patients' preoperative expectations, health literacy, social support, baseline pain catastrophizing, or subtle differences in surgeon experience could still influence both treatment response and self-reported outcomes. Third, the relatively small sample size (*n* = 120) limits statistical power to detect modest but clinically meaningful effects and precludes robust subgroup analyses. Finally, as a single-center study, the generalizability of findings to other settings—with different patient populations, nursing staffing ratios, or institutional resources—remains uncertain. Despite these limitations, our results highlight the crucial role of comprehensive nursing interventions in adenomyosis management, achieving dual improvements in patients' physical and psychological wellbeing through multifaceted care, thereby enhancing overall nursing outcomes. This model demonstrates practicality and broad applicability, offering valuable guidance for clinical practice. Future prospective, multicenter, randomized controlled trials with larger sample sizes are warranted to confirm these associations and establish the causal efficacy of comprehensive nursing protocols.

In conclusion, comprehensive nursing intervention, through multidimensional personalized measures, plays a significant role in the treatment of patients with adenomyosis. It effectively alleviates postoperative pain, improves sexual quality, and reduces the psychological burden and sexual dysfunction caused by the disease through psychological counseling and sexual health guidance, thereby enhancing overall quality of life. Moreover, comprehensive nursing intervention shortens postoperative recovery time, increases nursing satisfaction, and further improves patients' treatment adherence. Taken together, our findings, which are consistent with recent evidence from studies on perioperative recovery and sexual function following hysterectomy, suggest that structured, holistic nursing is an important modifiable factor associated with more favorable recovery trajectories in this patient population. This study demonstrates that the comprehensiveness and personalization of nursing interventions are key factors in improving care outcomes. Applying comprehensive nursing in the treatment of adenomyosis effectively eliminates patients' tension and fear, placing them in an optimal state for treatment. This not only improves therapeutic efficacy and shortens treatment duration but also enhances the quality of care and patient satisfaction, providing a valuable reference for clinical nursing practice.

## Data Availability

The original contributions presented in the study are included in the article/supplementary material, further inquiries can be directed to the corresponding author.
